# Early periprosthetic infection: dilution, jet dilution or local antibiotics. Which way to go? A meta-analysis on 575 patients

**DOI:** 10.3205/iprs000147

**Published:** 2020-10-28

**Authors:** Tilman Vees, Gunther O. Hofmann

**Affiliations:** 1 Clinic of Trauma, Hand und Reconstructive Surgery, Friedrich-Schiller-University Jena, Germany; 2 Department for Septic and Reconstructive Surgery, Clinic for Trauma and Reconstructive Surgery, BG-Kliniken Bergmannstrost, Halle (Saale), Germany

**Keywords:** prosthesis-related infections, anti-bacterial agents, therapeutic irrigation

## Abstract

**Objective:** Periprosthetic infections (PPI) after total hip and total knee arthroplasty (THA, TKA) are subdivided into early and late infections. Early PPIs are defined as the occurrence of infection within 6 weeks following the primary surgery. Aim of therapy in early PPI is the retention of the prosthesis using dilution, jet dilution or local antibiotics. However, as of yet, no evidence is available supporting these procedures. The aim of this study was to evaluate their success rates.

**Methods:** We conducted a systematic literature review of studies reporting on early PPI. Clinical trials published after 1990 that reported success or failure rates as the primary outcome were included. A meta-analysis using the Scheffé-Test showed if there are any advantages of single treatment concepts.

**Results:** We identified 575 patients over 10 studies. Success rates were diverse: Undergoing dilution without jet lavage revealed treatment success in 49.48%, using jet dilution increased the success rate to 78.26%. Local antibiotics were successfully used in 55% of the cases. The meta-analysis compared the three interventions and showed no significant difference in using dilution, jet dilution or local antibiotics. Even combining local antibiotics and dilution/jet dilution does not provide significantly higher success rates.

**Conclusion:** Previous studies showed differences in methods and results, however pooling the data of these studies for our meta-analysis didn’t show significant advantages. We therefore conclude that studies conducted until thus far cannot provide any recommendation as to whether using dilution, jet dilution, local antibiotics or any combination of three is better for treating early PPI cases.

## Zusammenfassung

**Ziel:** Periprothetische Infektionen (PPI) nach Implantation einer Hüft- und Kniegelenkendoprothese (THA, TKA) werden in frühe und späte Infektionen unterteilt. Frühe PPI sind definiert als das Auftreten einer Infektion innerhalb von 6 Wochen nach der primären Operation. Ziel der Therapie bei frühen PPI ist, die Prothese unter Verwendung von Spülung, Jet-Lavage oder lokaler Antibiotikatherapie zu erhalten. Bisher besteht jedoch noch keine Evidenz für den Einsatz dieser Verfahren. Ziel dieser Studie ist es, ihre Erfolgsraten zu bewerten.

**Methoden:** Wir führten eine systematische Literaturrecherche durch. Eingeschlossen wurden nach 1990 veröffentlichte klinische Studien, die Erfolgs- oder Misserfolgsraten in der Behandlung von frühen PPI angegeben. Mittels Metaanalyse und Scheffé-Test wurde untersucht, ob einzelne Behandlungskonzepte Vorteile bieten.

**Ergebnisse:** Es wurden 575 Patienten in 10 Studien identifiziert. Die Erfolgsraten waren je nach verwendetem Verfahren unterschiedlich: Die Spülung ohne Jet-Lavage zeigte einen Behandlungserfolg von 49,48%, wobei der Einsatz einer Jet-Lavage die Erfolgsrate auf 78,26% erhöhte. In 55% der Fälle wurden lokale Antibiotika erfolgreich eingesetzt. Die Metaanalyse verglich die drei Interventionen, wobei sich kein signifikanter Unterschied in der Verwendung der drei Methoden zeigte. Selbst die Kombination von lokalen Antibiotika und Spülung/Jet-Dilution führt nicht zu signifikant höheren Erfolgsraten.

**Fazit:** In früheren Studien zeigten sich unterschiedliche Konzepte der Methodik sowie voneinander abweichende Ergebnisse. In der vorliegenden Arbeit konnten keine signifikanten Vorteile für eine der Therapieformen gezeigt werden. Wir schließen daraus, dass aus den bisher durchgeführten Studien keine evidenzbasierte Empfehlung abgegeben werden kann, ob die Verwendung einer Spülung, Jet-Dilution oder der Einsatz lokaler Antibiotika respektive deren Kombination einen Therapievorteil bei frühen PPI zeigen.

## Introduction

Periprosthetic infection (PPI) is one of the most feared and devastated complications following total joint replacement [1]. Fortunately, less than 1% of total hip and knee arthroplasties (THA, TKA) in the US result in PPI today [2].

Classification of PPI depends on the activity of infection (acute, chronic, quiescent) [3] and time of infection in reference to the primary surgery (early, late): Zimmerli et al. divided PPI into early (up to 3 months after primary surgery), delayed (3 to 24 months) and late infection (over 24 months) [4]. Cut-off for early infection is as well set at 4 to 6 weeks after primary surgery [5], [6].

In early PPI with a well-fixed prosthesis and without sinus tract, irrigation and debridement may be performed within 4 weeks after prosthesis implantation. Removal of the prosthesis is not necessary, but mobile parts should be replaced [7]. This should improve infection control since the drug-resistant biofilm is established later [7], [8]. Additionally, local antibiotics can provide up to 1,000 times higher concentration at the place of infection than systemic antibiotics. This can be reached without exposing the patient to toxic serum levels [9]. Furthermore, dilution with iod, saline or antibiotic solution is a common treatment for PPI. Saline solutions are more common, as the iodic-solutions lose their antiseptic effect in presence of proteins [10]. Dilution can be either carried out with low- or high pressure. Although there is no definition, flow in low pressure dilation ranges between 1 and 15 psi and between 35 to 70 psi in high pressure dilution [11].

Since no evidence-based treatment protocol exists for early PPI, our meta-analysis seeks to determine if any recommendations can be given for treating early PPI. 

## Methods

A database enquiry on PubMed identified relevant publications. Search terms were “bone and joint infection”, “osteomyelitis”, “endoprosthetic infection”, “prosthesis-related infection”, “jet lavage”, “irrigation treatment” and “local antibiotics”. The search was updated periodically. Publications found twice were excluded. 

We selected articles that were published as an original clinical trial focusing on the treatment of PPI but limited the study articles published since 1990 in English or German. Their relevance was assessed through an examination of the titles and abstracts. Articles with no abstract electronically available were not included. We also excluded articles that did not carry out a clinical trial and/or described PPI as secondary observation.

Publications were organised and categorised using Citavi (Swiss Academic Software GmbH, Wädenswil, Switzerland). Full texts were analysed by study type, population size, population’s age and follow-up, surgical procedure (prosthesis retention vs. exchange arthroplasty), surgical interventions (debridement, (jet) dilution, local antibiotics and use of a spacer), use of systemic antibiotics and the individual outcome. In addition, if provided, the period of time between primary surgery, clinical manifest infection and intervention was recorded. A differentiation between early and late PPI was carried out, with early PPI defined as a display of clinical and laboratory sign of infection within 6 weeks of primary surgery. 

The meta-analysis considered only studies reporting about early PPI and providing success or failure rates as their primary outcome. Successful treatment was the absence of infection and no need of further revision surgery. We operated on the assumption that one patient can either be treated successfully or unsuccessfully. Therefore, failure rates were converted into success rates by subtracting failed cases from the study’s cohort. Furthermore, to avoid underestimating studies with small populations or overestimating those with large ones we assembled a homogeneous sample size by reducing study populations to 20 participants. Success rates were not modified.

Patients were divided into four groups depending on the surgical procedure. Type of dilution was separated by defining A, B and C (Table 1 (Tab. 1)).

For the variance analysis using IBM SPSS Statistics (IBM, Ehningen, Germany), we observed the infection type in addition to the surgical procedure of each patient. The mean success rates as well as the number of patients in each group were calculated. 

The Scheffé-Test determined whether the variance analysis showed a significant difference between groups 1 to 4 (F Prob.<0.05). The significance level was 0.05. Additionally, the Chi-square test by Van der Waerden compared A, B and C of group 1 and 3. The significance level was 0.05.

In order to ensure adequate quality of this meta-analysis, the investigations were carried out in accordance with the PRISMA guidelines [12].

## Results

The first database enquiry on 13^th^ May 2015 identified 1,862 articles. We continuously updated our search, with last search conducted on 7^th^ May 2017 revealing 2,156 results.

**Figure 1:**
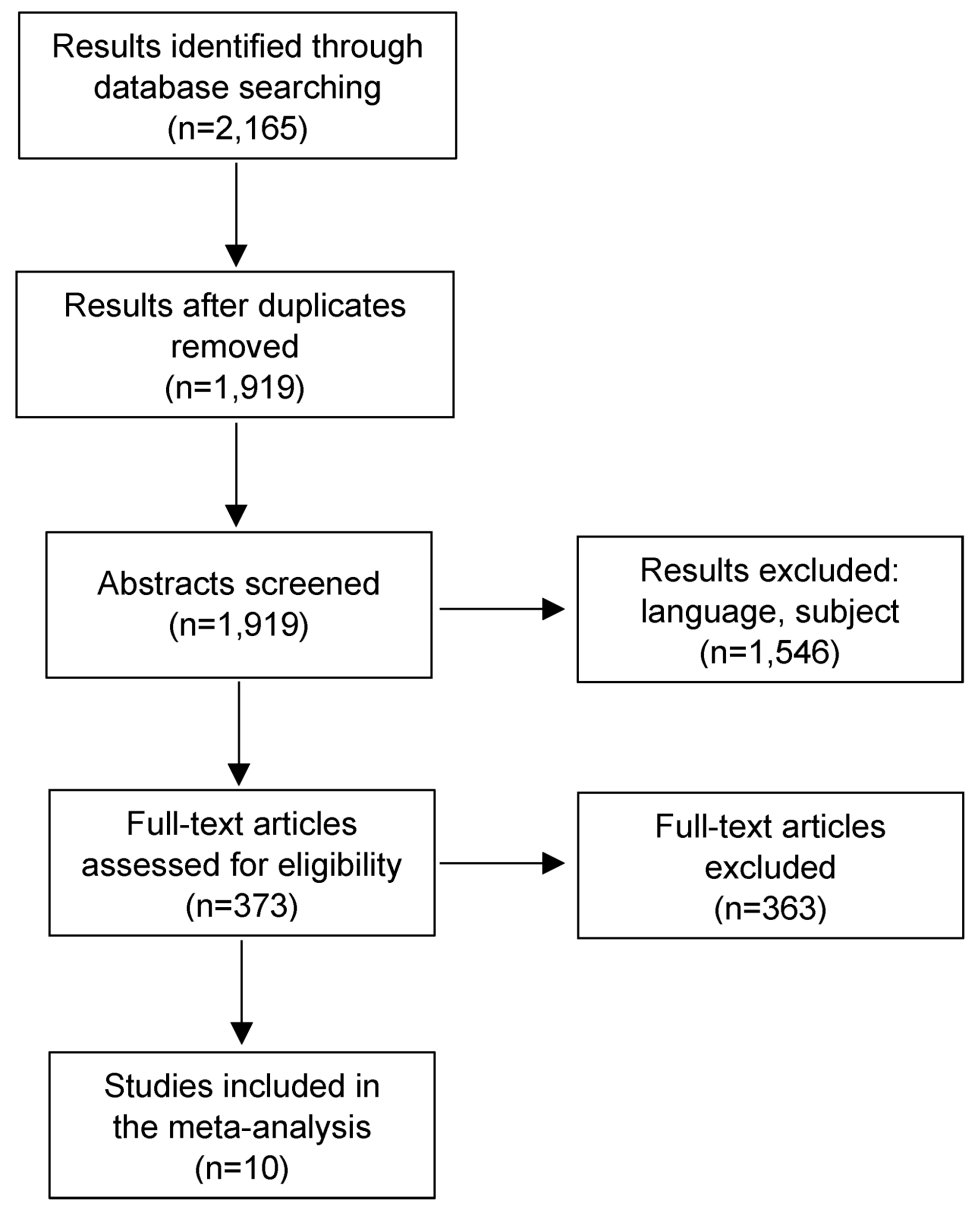
Flow diagram (PPI: periprosthetic infection)

The analysis and evaluation of the articles were carried out by the author. Inclusion and exclusion criteria were applied by going through the titles and abstracts. This reduced the number of relevant publications from 1,919 to 373. Furthermore, articles were excluded because of reporting about outcome rather than success or failure rates as well as late or unspecified infection. At last, 10 publications with a total of 575 patients remained for a meta-analysis (Figure 1 (Fig. 1)).

Although our database enquiry searched studies published since 1990, our search did not yield a single study with relevant data from before 2006. The 10 selected articles were published between 2006 and 2013. All studies reported on THA and TKA, except Cobo et al.’s study on one shoulder arthroplasty. The study designs were diverse. Most of the clinical trials were carried out retrospectively (n=9). Sample sizes varied from 3 to 145 participants. Most patients were treated by only dilution (414 patients in 7 studies). Groups 2 to 4 each contain only one study (Table 2 (Tab. 2)). Prosthesis retention was the chosen procedure in 7 studies (373 patients). 202 patients in 3 studies underwent one-stage exchange arthroplasty [5], [13] or both prosthesis retention and two-stage exchange arthroplasty [6]. 

Statistical analysis showed successful treatment by non-jet dilution in 55.84%. By using jet dilution treatment alone, a success rate of 79.31% was achieved. Using local antibiotics without dilution showed success in 53.33% of the cases (Table 3 (Tab. 3)). However, the comparison by Chi-square test showed no significant difference between these groups. 

Analysing statistically reduced values (Table 4 (Tab. 4)) showed success in 49.48% of cases of non-jet dilution, 78.26% of cases treated with jet dilution and 55% of cases of local antibiotics. Combining non-jet dilution and local antibiotics was successful in 45% of the patients. Carrying out neither local antibiotics nor dilution yielded the success rate of 70% (group 4). Again, no significant advantages were found. Facing the group of patients with early PPI diagnosed within 4 weeks after primary surgery brought few data, so no data analysis could be performed.

## Discussion

The cause of PPI include perioperative surgical infections, intraarticular injections or haematogenous infections since introducing foreign material increases the appearance of pathogens [14]. Many pathogens are possible. This may be due to the biofilm that develops on the surface of the prosthesis. The bacterial adhesion is enhanced within the biofilm because pathogens are protected against antibiotics and the immune system. Fractures, especially open ones, implant surfaces, and external fracture fixations are examples of situations that are known to enhance bacterial adhesion. These conditions, if left untreated, may lead to a biofilm formation and osteomyelitis [15], [16]. Additionally, pathogens found in biofilms have lower replication rates which make them less sensitive against antibiotics [17]. *S. aureus* or *S. epidermidis* are responsible for 60–90% of infections and 25% of PPI cases are caused by more than one pathogen (mixed infection) [18]. High virulent pathogens (*S. aureus*, Streptococcus, Enterococcus) are especially responsible for early infection, low virulent pathogens (Coagulase-negative Staphylococcus, Propionibacterium acnes) for delayed PPI [7]. Treatment of PPI is difficult and often long lasting. This is a great burden to patients and results in high health care costs. Because of long hospitalisation time and additional surgery, one single PPI can incur costs of about 55,000 € [18]. In the United States, costs of hospitalisation were 1.76 times higher in infected than in uninfected THA [2]. According to the German endoprosthetic register (2017), “infection” was listed as the reason in 18.9% follow-up surgeries after primary implantation of THA and in 22.3% follow-up surgeries after primary implantation of TKA [19].

Prosthesis retention is the most common way to treat early PPI. Systemic or local antibiotics in addition to dilution can be used. There’s an agreement that DAIR (debridement, antibiotics, irrigation and prosthesis retention) is generally accepted for early PPI treatment [9], [20]. However, until today, there are no guidelines as to what calls for various treatment strategies. Renz et al. recommended prosthesis retention if the infection occurs within 4 weeks after implantation [7]. The Clinical practice guidelines by the Infectious Diseases Society of America sees good evidence (A-II) for prosthesis retention if the duration of the infection symptoms is less than 3 weeks [21]. The upper cut off for early PPI is unclear. Dzaja et al. saw the upper limit for postoperative infection at 4 weeks after primary surgery [6], Hansen et al. considered the limit between 4 and 6 weeks [5]. Ruchholtz et al. defined every PPI occurring later than 30 days after implantation as a chronic infection [3]. The shift from early to late PPI is often marked at 3 months [4], [20], [22], [23]. Observations showed that patients have problems describing the duration of symptoms. This makes it difficult to learn about the onset time of infection. One reason could be the diversity of clinical presentations. However, correct classification and the beginning of a suitable therapy is important for therapeutic success. Studies considered in our meta-analysis reported on success rates ranging from 16% to 100% for different treatment protocols (Table 5 (Tab. 5)) [24].

Local antibiotics were first used in 1970 by Buchholz and Engelbrecht by combining them with polymethyl metacrylate (PMMA) [25]. Based on their concept, in 1977 Klemm described antibiotic beads consisting of PMMA and gentamicin as an alternative treatment to dilution [26]. In 1993, Blaha et al. compared local (Septopal^®^) versus conventional systemic antibiotic therapy and showed that adverse experiences were higher in conventional antibiotic treatment [27]. Today, local antibiotics can be processed in cement, beads or sponges. Gentamicin sponges do not require removal, which is a necessary part of the procedure when using beads. Still both showed comparable results [9]. Gentamicin is a heat stable antibiotic agent which makes it suitable for the use in cement spacers [17]. This method has become the gold standard in treating implant-related infections by 2-stage revision. 

Dilution therapy can be performed with high or low pressure. Even if effectiveness of bacterial reduction depends on dilution pressure [28], both systems showed similar success rates in treating early PPI [11], [29]. High-pressure dilution systems can either be performed continuously or pulsatively. Both can get into deep wounds removing necrotic tissue thoroughly [29]. In 1978, Brown et al. showed that pulsatile jet dilution can reach a significant bacteria count reduction in contaminated rat wounds [30]. However, it is unclear if high pressure lavage may have disadvantages in bone healing and infection treatment: in vitro models showed bacterial seeding and bone architecture damage [31]. Moreover, ovine models showed that high pressure pulsatile lavage drives bacteria into deeper levels if used in tissue infection [11]. Using low pressure dilution likely increases the risk of reinfection [29].

Prosthesis retention with dilution with an antibiotic-laden saline solution and debridement was performed by Azzam et al. [32] in a single-centre study of 104 patients, suffering either from early PPI or presenting symptoms suggesting hematogenous PPI. Success rate was 44.2%. No statistically significant difference in regard to symptom duration was found after comparing success and failure groups. The author concludes that no time interval between onset of symptoms and debridement has been established yet [32].

Bradbury et al. [24] reviewed 19 patients with acute infection of the knee by methicillin-resistant *Staphylococcus aureus*. Treatment protocol consists of open debridement and dilution with prosthesis retention and systemic antibiotics. Treatment failed in 84% of the cases. The author suspects that the average age of the cohort (72 years) may reduce the cohort’s prospects of success [24].

A comparable, but prospectively designed study was carried out by Cobo et al. [33]. In 117 patients suffering from PPI in hip, shoulder or knee, dilution and debridement was applied with prosthesis retention and systemic antibiotics. Only occurrence of PPI within 30 days after arthroplasty were included. Success rate was a much-improved 58%. The author criticised the variety of definitions describing success. As there is no standard protocol, treatment success is difficult to compare [33].

Dzaja et al. [6] reported on 145 patients in 2015. 69% of the cases were successful across both groups by using dilution with either 2-stage revision or prosthesis retention. Also, Fehring et al. [8] carried out irrigation and prosthesis retention in a multicentric retrospective analysis. Following 84 patients with early PPI in THA and TKA, success was reached in 63% of the cases. Whereas the above-mentioned studies all used local or systemic antibiotics, Dzaja et al. and Fehring et al. did not make use of these treatments. 

Hansen et al. [5] and Wick et al. [13] presented retrospective studies, treating patients with one-stage exchange arthroplasty without using dilution. Hansen et al. followed 27 patients and reported success in 70%. Only one stage exchange arthroplasty with systemic antibiotic therapy was carried out. Wick et al. treated 30 patients with antibiotic laden cement and reached a 55% success rate.

In 2006, Lehner et al. [34] reported on 3 patients treated with jet dilution with prosthesis retention. Infection control was reached in all 3 patients. Also, Sukeik et al. [35] treated 26 patients with jet dilution and prosthesis retention. In this study, 77% of infections could be controlled. Differences showed up in the age of patients. While Lehner et al.’s eldest patient was 75 years old, Sukeik’s patients were up to 86 years old. 

In addition, Van Kleunen et al. [36] reported on patients treated by prosthesis retention. However, dilution was carried out without pulsed function. The studies of 18 patients yielded a 72% success rate. 

Based on reduced study groups (see Methods section) we can record that the use of local antibiotics provides higher success rates than using non-jet dilution (55%; 49.48%). Regarding jet dilution we observe that, again for reduced study groups, jet dilution is superior to local antibiotics (78.26%; 55%).

Carrying out either non-jet dilution or local antibiotics seems to be superior to combining both (49.48%; 55%; 45%). Additionally, using none of them showed superior results than combined or single use. Using only jet dilution seems to be favourable in comparison to combined use of jet dilution and local antibiotics (78.26%; 70%). Those comparisons are tendencies. In none of those comparisons, significant differences were found.

In our analysis, we can see little benefit in using local antibiotics. This is remarkable. One reason could be low concentration of the local antibiotic agent. A concentration lower than the minimal inhibition concentration (MIC) may increase the chance of infection persistence or recurrence [37].

Not taken into consideration was the use of systemic antibiotics. 2 studies went without systemic antibiotics and are part of group 1. Within this group, an above-average success rate of infection treatment was published. Therefore, it is unclear how systemic antibiotic therapy influences our results.

Important in therapy of PPI seems to be the formation of the biofilm. Once it has been established, it offers a perfect environment for bacterial growth. It can be formed within 72 hours. Early surgical intervention is necessary for infection control. Therefore, therapy should be performed promptly and aggressively [10].

Not taken into consideration in our meta-analysis was the type of debridement. Debridement is not a well-defined procedure and there’s a lack of standardisation. We noticed that in 6 studies the debridement process is described [5], [32], [33], [34], [35], [36]. Furthermore, change of poly ethylene parts was not to be forced. 

Our meta-analysis is the first investigating the use and impact of (jet) dilution and local antibiotics. We showed that the use of a jet dilution compared to conventional irrigation achieves higher success rates. However, there is a lack of data to get reliable results; our results are partially based on single studies. Additionally, most studies are limited by small study populations. Furthermore, variance in studies is large. The lack of standardisation in naming and treating PPI poses a challenge for comparing published data. To learn more about effectiveness of single treatment protocols, large prospective studies and standardised treatment protocols would be necessary.

## Conclusions

PPI is a clinically relevant issue and dilution, jet dilution as well as local antibiotics provide different success rates in infection treatment. Jet dilution seems to be more effective in comparison to conventional irrigation or local antibiotics. However, there exists no significant difference. We conclude that – based on studies until today – we cannot provide any recommendation for the treatment of early PPI.

## Abbreviations

- PPI: periprosthetic infections
- THA: total hip arthroplasty
- TKA: total knee arthroplasty
- PMMA: polymethyl metacrylate
- MIC: minimal inhibition concentration

## Notes

### Competing interests

The authors declare that they have no competing interests.

## Figures and Tables

**Table 1:**
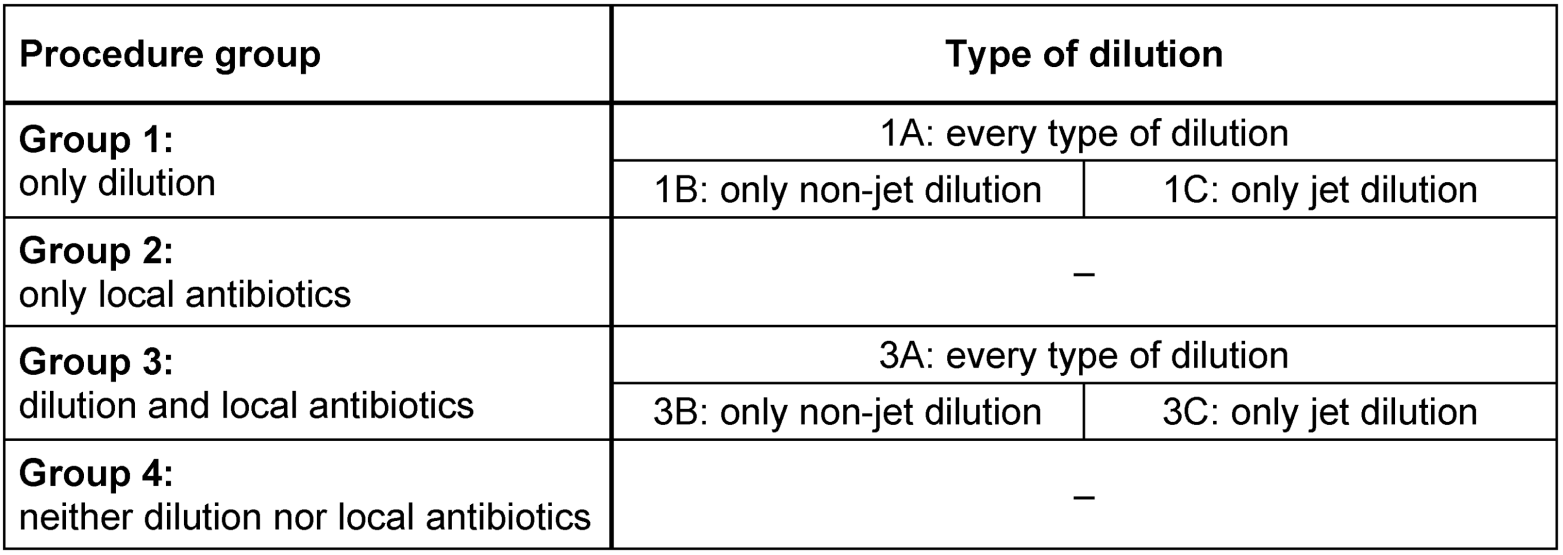
Groups and procedures

**Table 2:**
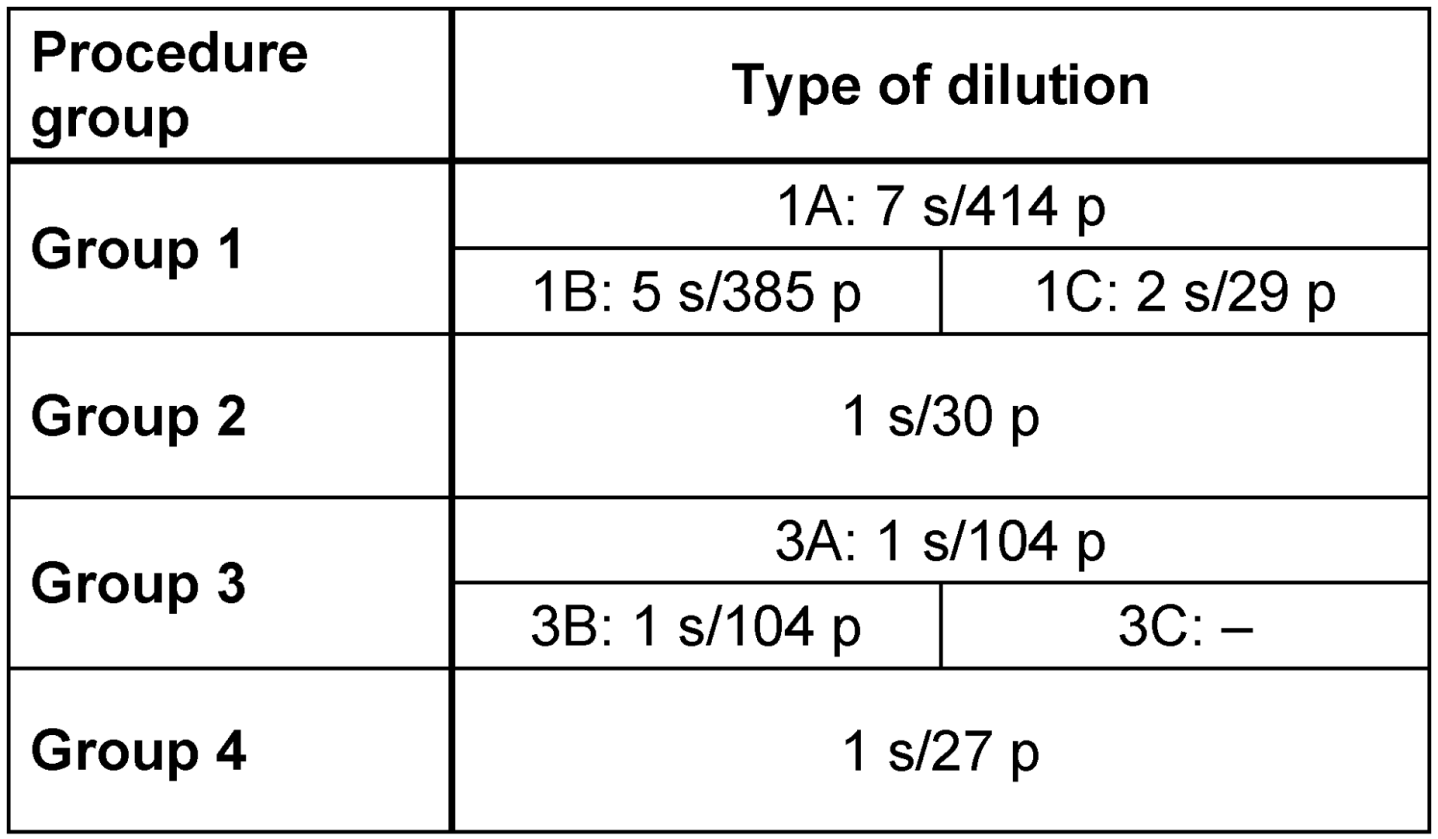
Number of studies (s) and patients (p) of each group

**Table 3:**
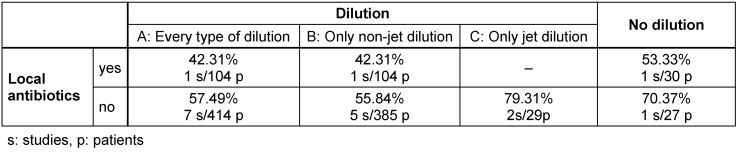
Success rate of dilution, jet dilution and local antibiotics in early PPI

**Table 4:**
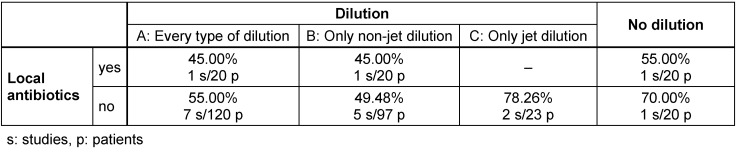
Combining dilution and local antibiotics in early PPI (reduced values)

**Table 5:**
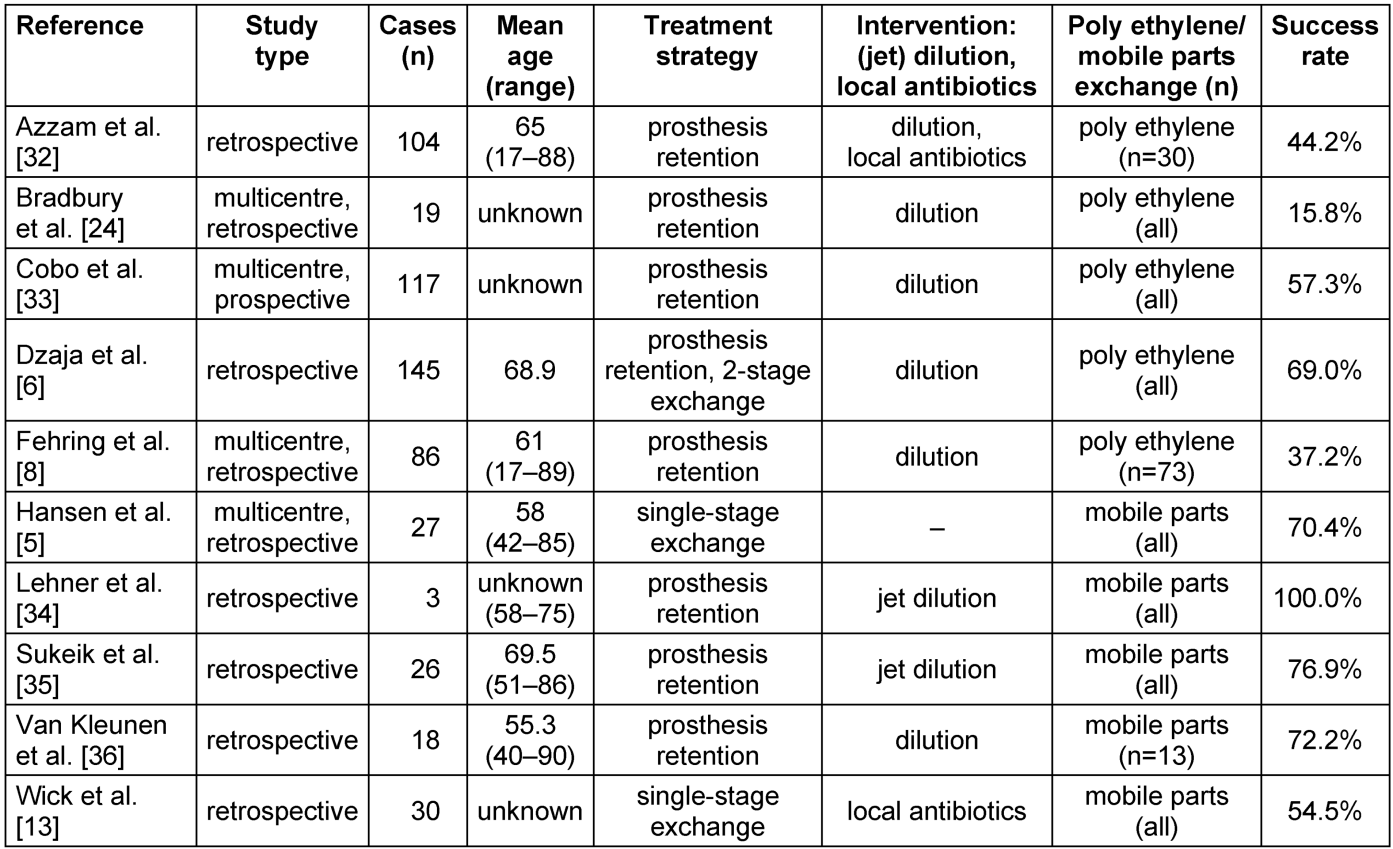
Studies of the meta-analysis
